# Biosynthetic gene profiling and genomic potential of the novel photosynthetic marine bacterium *Roseibaca domitiana*

**DOI:** 10.3389/fmicb.2023.1238779

**Published:** 2023-09-29

**Authors:** Giuliano Gattoni, Fabiana Di Costanzo, Rafael R. de la Haba, Ana B. Fernández, Shaday Guerrero-Flores, Nelly Selem-Mojica, Antonio Ventosa, Paulina Corral

**Affiliations:** ^1^Department of Biology, University of Naples Federico II, Naples, Italy; ^2^Department of Microbiology and Parasitology, Faculty of Pharmacy, University of Sevilla, Sevilla, Spain; ^3^Institute for Multidisciplinary Research in Applied Biology, Public University of Navarre, Pamplona, Spain; ^4^Research & Development Department, Bioinsectis SL, Navarre, Spain; ^5^Centro de Ciencias Matemáticas, Universidad Nacional Autónoma de México UNAM, Morelia, Mexico

**Keywords:** biosynthetic profiling, photosynthetic bacteria, bacteriochlorophyll, genome mining, *Roseibaca*, biosynthetic gene cluster (BGC)

## Abstract

Shifting the bioprospecting targets toward underexplored bacterial groups combined with genome mining studies contributes to avoiding the rediscovery of known compounds by revealing novel, promising biosynthetic gene clusters (BGCs). With the aim of determining the biosynthetic potential of a novel marine bacterium, strain V10^T^, isolated from the Domitian littoral in Italy, a comparative phylogenomic mining study was performed across related photosynthetic bacterial groups from an evolutionary perspective. Studies on polyphasic and taxogenomics showed that this bacterium constitutes a new species, designated *Roseibaca domitiana* sp. nov. To date, this genus has only one other validly described species, which was isolated from a hypersaline Antarctic lake. The genomic evolutionary study linked to BGC diversity revealed that there is a close relationship between the phylogenetic distance of the members of the photosynthetic genera *Roseibaca, Roseinatronobacter*, and *Rhodobaca* and their BGC profiles, whose conservation pattern allows discriminating between these genera. On the contrary, the rest of the species related to *Roseibaca domitiana* exhibited an individual species pattern unrelated to genome size or source of isolation. This study showed that photosynthetic strains possess a streamlined content of BGCs, of which 94.34% of the clusters with biotechnological interest (NRPS, PKS, RRE, and RiPP) are completely new. Among these stand out T1PKS, exclusive of *R. domitiana* V10^T^, and RRE, highly conserved only in *R. domitiana* V10^T^ and *R. ekhonensis*, both categories of BGCs involved in the synthesis of plant growth-promoting compounds and antitumoral compounds, respectively. In all cases, with very low homology with already patented molecules. Our findings reveal the high biosynthetic potential of infrequently cultured bacterial groups, suggesting the need to redirect attention to microbial minorities as a novel and vast source of bioactive compounds still to be exploited.

## 1. Introduction

Microbial diversity and all its metabolic potential are being explored by massive sequencing techniques and the use of genome mining strategies. Metagenomic studies and further identification of specialized metabolite biosynthetic gene clusters (BGCs) have demonstrated that we are obviating most of the prokaryotic diversity and, consequently, all its metabolic potential (Scherlach and Hertweck, 2021; Paoli et al., 2022). Most of what is known to date about microbial diversity is restricted to genomic sequences, which are reconstructed as metagenome-assembled genomes (MAGs). Thus, the vast majority of the bacterial community remains uncultivated. In some environments, such as the Mediterranean Sea, a large proportion of its microbial population is represented by members of the phylum *Pseudomonadota*, which in turn encompasses groups still to be discovered and that count as minorities (Haro-Moreno et al., 2018). It is also well known that culture failure is due to many bacteria thriving solely in their natural environment and community as ecto/endosymbionts (Yang et al., 2022) or as part of complex structures as microbial mats (Chen et al., 2020).

Recognizing the challenges of culturing new taxa, new strategies have been developed and implemented to address the “non-cultivable” fraction and overcome this limitation. Among them, the exploration of extreme and poorly explored environments, such as the seabed (Feling et al., 2003; Eustáquio et al., 2011; Jensen et al., 2015), the employment of *in situ* cultivation techniques through isolation devices (Berdy et al., 2017; Lodhi et al., 2018), and other isolation approaches based on microbial co-culture (Stewart, 2012; Sánchez-Andrea et al., 2018), stand out. All of them have enabled the isolation of new taxa that are currently being explored at a biotechnological level, mainly in the pharmacological industry (Bauman et al., 2022). In addition to these methods, we have recently proposed the diel and seasonal sampling as a strategy to increase the probability of isolating new marine microorganisms. These studies considered dynamic phenomena, such as diel vertical migration (DVM) and seasonality with the consequent microbial redistribution, allowing the isolation of the new bacterium *Leeuwenhoekiella parthenopeia*, belonging to the rare biosphere and, interestingly, with the capacity to inhibit the viability of tumor cells *in vitro* (Gattoni et al., 2023).

The primary resource to establish the biosynthetic potential of a bacterium is the genome mining strategy, whose application highly contributes to the prevention of the rediscovery of already-known natural products (Ward and Allenby, 2018; Gavriilidou et al., 2022). The biosynthetic capacity is assessed by predicting the main classes of BGCs, such as polyketide synthase (PKS), non-ribosomal peptide synthase (NRPS), ribosomally synthesized and post-translationally modified peptides (RiPPs), and RiPP recognition element (RRE), all involved in the secondary/specialized metabolic pathways targeted to synthesize bioactive compounds of biotechnological interest (Medema et al., 2015; Kloosterman et al., 2020). A homology search with known BGCs from other organisms encoding the synthesis of chemically deciphered and experimentally characterized compounds is a common approach to estimating the metabolic capacity of the bacterium of interest as a potential source of lead candidates before investing more time and money in further biomedical and biotechnological applications (Doroghazi et al., 2014; Pan et al., 2017; Parkinson et al., 2018; Belknap et al., 2020).

In the present study, we determined the biosynthetic profile and the genetic potential of a novel photosynthetic marine bacterium, designated as *Roseibaca domitiana*, which was isolated after diel and seasonal sampling from the Tyrrhenian Sea coast, as previously reported for *Leeuwenhoekiella parthenopeia*. This pink-pigmented bacterium is phylogenetically closely related to the only representative species of the genus *Roseibaca, Roseibaca ekhonensis*, a bacterium originally isolated from a saline lake in Antarctica (Labrenz et al., 2009). However, the new isolate showed the closest similarity with an uncharacterized cultured species, “*Roseibaca calidilacus*,” which was isolated from a microbial mat of the heliothermal hypersaline lake Hot Lake (Washington, USA) (Romine et al., 2017). Those two related cultivated species of the genus *Roseibaca* are derived from extreme aquatic environments. Thus, *Roseibaca domitiana* constitutes the first representative of this genus isolated from a marine environment. Taxonomically, this new bacterium belongs to the genus *Roseibaca*, classified in the family *Paracoccaceae* (Liang et al., 2021; Göker, 2022), order *Rhodobacterales*, class *Alphaproteobacteria*, and phylum *Pseudomonadota* (Oren and Garrity, 2021). In particular, the members of the family *Paracoccaceae* are widely distributed in the marine environment, representing up to 30% of the bacterial community in some aquatic environments (Lamy et al., 2011; Sato-Takabe et al., 2016; Simon et al., 2017). The ability to synthesize pigments, such as bacteriochlorophyll, is an intrinsic characteristic of some genera of purple photosynthetic bacteria, especially of Aerobic Anoxygenic Phototrophs (AAP) (Madigan and Jung, 2009), whose biosynthetic potential remains unexplored.

The comparative genomic study carried out allowed us to determine the biosynthetic capacity of *Roseibaca domitiana* with respect to closely related members of the family *Paracoccaceae* from an evolutionary perspective and based on the novelty of BGCs. We provide the first insights into the lack of correlation between the phylogenetic distance and the BGC profiles of members of this family. Specifically, we highlight and discuss the novel BGCs identified in *Roseibaca domitiana* as potential synthesizers of compounds for plant improvement and antitumor agents, proposing this species as a promising bioresource in the agricultural and pharmaceutical industries.

## 2. Materials and methods

### 2.1. Sampling and isolation

The strain V10^T^ was isolated from the littoral Domitian Coast, Italy (40°54′50.2 “N 14°01′26.2” E), during a diel and seasonal study of the Tyrrhenian Sea. The seawater samples were taken during the fall and winter of 2020–2021 and subsequently processed for 2 h on media prepared with the same filtered natural seawater. Salinity, pH, conductivity, particulate [total dissolved solids (TDSs)], and temperature were measured *in situ* with a refractometer and a digital multiparameter pH/EC/TDS/TEMP (Hanna HI-9812-51). The isolation was performed by extinction dilution technique on solidified seawater oligotrophic medium (SWOM). The plates were aerobically incubated at room temperature (~21°C) following the light day cycle, and the colonies were visible after 6 days of incubation. Following five subculture passages, the strain V10^T^ was able to grow on the synthetic medium, marine agar (MA) 2216 (Difco). For long-term conservation, the strain V10^T^ was grown in marine broth (MB) for 72 h and stored at −80°C in cryovials with 40% (v/v) of glycerol.

SWOM composition (g/L of natural seawater): casamino acids, 0.5; yeast extract, 1.0; tryptone, 1.0; and agar, 15.0. Supplements: glycerol, 0.1 M; mineral solution, 500 μL/L; and vitamin solution, 500 μL/L. All supplement solutions were sterilized by filtration and added after autoclaving. The vitamin solution composition (mg/L) was as follows: biotin, 4.0; folic acid, 4.0; pyridoxine-HCl, 20.0; riboflavin, 10.0; thiamine-HCl 2H_2_O, 10.0; nicotinamide, 10.0; D-Ca-pantothenate, 10.0; vitamin B_12_, 0.20. The volume was brought up to 1,000 mL using distilled water. The solution was stored in the dark at 4°C.

### 2.2. Molecular identification of the strain V10^T^

The strain V10^T^ was initially identified using 16S rRNA gene sequencing after PCR amplification. Universal primers 27F and 1492R were used in a 40 μL master mix that included a 5 × reaction buffer, which contains dNTPs, MgCl_2_, and enhancers. MyTaq *Taq* DNA polymerase from Bioline and nuclease-free water were also part of the mix. The PCR procedure involved an initial denaturation at 95°C for 5 min, followed by 25 cycles that included 30 s at 94°C, 30 s at 50°C, and 90 s at 72°C. A final extension was carried out for 10 min at 72°C. Afterward, the PCR product was verified through 1% agarose gel electrophoresis and purified using a GeneJET PCR purification kit from Invitrogen. The sequencing was conducted by Stab Vida in Portugal using the Sanger method. Finally, sequence quality control and assembly were conducted using 4Peaks v.1.8 and ChromasPro v.2.1.10 software, respectively.

The assembled 16S rRNA gene sequence from strain V10^T^ was compared with the EzBioCloud database (Yoon et al., 2017) using the 16S-based ID service. The sequence identity was also calculated using NCBI BLAST (RRID: SCR_004870) against the NCBI nr/nt nucleotide database (RRID: SCR_004860) restricted to sequences from type material but also including uncultured/environmental sample sequences.

### 2.3. Genomes sequence retrieval and estimation of Overall Genome Relatedness Indices

For genome sequencing, high-quality DNA was obtained using the DNeasy UltraClean kit (QIAGEN, Hilden, Germany), following the protocol for Gram-negative bacteria. DNA purity and quantification were determined by fluorometry using a Qubit assay kit for double-stranded DNA broad range (dsDNA BR, Invitrogen) and measured with a Qubit 4 fluorometer (Invitrogen, MA, USA).

The genome of strain V10^T^ was sequenced using the Illumina NovaSeq 6000 system 2 × 150 PE (Novogene Co., UK). Raw read quality was assessed using FastQC (RRID: SCR_014583) v.0.11.9, and those containing adapters and low-quality bases (Q ≤ 20) were removed with Trimmomatic (RRID: SCR_011848) v.0.36 (Bolger et al., 2014). *De novo* assembly of the filtered reads was performed using SPAdes (RRID: SCR_000131) v.3.15.4 (Bankevich et al., 2012) and SOAPdenovo2 (RRID: SCR_005503) (Luo et al., 2012). Assembly quality was estimated with QUAST (RRID: SCR_001228) v.5.1.0rc1 (Gurevich et al., 2013), and its completeness and contamination were assessed using CheckM (RRID: SCR_016646) v.1.0.5 (Parks et al., 2015). The curated genome sequence was annotated using the NCBI Prokaryotic Genomes Automatic Annotation Pipeline (PGAP) (RRID: SCR_021329) (Tatusova et al., 2016) and deposited in GenBank/EMBL/DDBJ under accession number JALZWP000000000.

For comparative genomic studies, 36 genome sequences of cultivated species closely related to the strain V10^T^ were obtained from NCBI GenBank (Supplementary Table 1). This genomic comparison was performed between strain V10^T^ and type strains of validly published species names. Additionally, the study included the genomes of non-type strains of closely related cultivated species: “*Roseibaca calidilacus*” HL-91, *Roseibaca* sp. Y0-43*, Roseinatronobacter* sp. HJB301, and “*Natronohydrobacter thiooxidans”* AH01.

To circumscribe the strain V10^T^ as a new taxon, several Overall Genome Relatedness Indices (OGRIs) (Chun and Rainey, 2014) were estimated. The average nucleotide identity based on BLASTn+ (RRID: SCR_001598) (denoted as ANIb) was calculated using pyANI v.0.2.12 software (Pritchard et al., 2016). The digital DNA–DNA hybridization (dDDH) was calculated using the Genome-to-Genome Distance Calculator (GGDC) v.3.0, with formula *d*_4_ (a.k.a. GGDC formula 2) (Meier-Kolthoff et al., 2013). The results were evaluated based on the cutoff for species boundaries: ANI, 95–96% (Konstantinidis and Tiedje, 2005; Goris et al., 2007; Richter and Rosselló-Móra, 2009; Chun and Rainey, 2014), and dDDH, 70% (Auch et al., 2010).

### 2.4. Phylogenetic and phylogenomic reconstruction

The 16S rRNA gene-based phylogeny was performed using the online tool Type Strain Genome Server (TYGS) (Meier-Kolthoff and Göker, 2019; Meier-Kolthoff et al., 2022), including, among others, the genome sequences of the strain V10^T^ and its most closely related species of the genus *Roseibaca*. Given the well-known inaccuracy of determining evolutionary relationships by using single locus phylogenies (Corral et al., 2018; de la Haba et al., 2018; Infante-Domínguez et al., 2020), a more detailed genome-based tree was inferred, as explained below.

Orthologous gene clusters present in all the genomes (core-genome dataset) were determined using all-vs.-all BLASTp+ (RRID: SCR_001010) comparisons among the translated coding sequences (CDS) of the annotated genomes under study, as implemented in the “anvi-pan-genome” script (options “–min-percent-identity 40 –minbit 0.5 –mcl-inflation 5 –use-ncbi-blast”) from Anvi'o suite v.7.1 (Eren et al., 2020). Then, single-copy core protein sequences were individually aligned with Muscle v. 3.8.1551 (Edgar, 2004). Alignment columns for each individual gene with more than 50% gaps or highly hipervariable were removed by means of the “alignment_pruner.pl” Perl script (https://github.com/novigit/davinciCode/blob/master/perl). Pruned alignments were finally concatenated into a super-matrix using the AMAS tool (Borowiec, 2016), which was further analyzed to estimate the best model and parameters of amino acid substitution via ModelFinder (Kalyaanamoorthy et al., 2017). The maximum-likelihood phylogenomic tree was generated using IQ-TREE v.2.2.0 (Minh et al., 2020), and the tree branch support was inferred using the ultrafast bootstrap approximation (Hoang et al., 2018).

For phylogenetic analyses based on photosynthetic genes, protein-coding sequences were predicted using Prodigal v.2.6.3 (Hyatt et al., 2010) and annotated with eggNOG-mapper v.2.1.9 (Cantalapiedra et al., 2021). Translated sequences of *pufLM, puhA*, and *bchXYZ* photosynthetic genes were aligned with Muscle v.3.8.31 (Edgar, 2021) and then concatenated with SeqKit v.2.3.0 (Shen et al., 2016). The best-fit model of amino acid sequence evolution was calculated using ProTest v.3.4.2 (Abascal et al., 2005). The maximum-likelihood tree (using the WAG model) with 1,000 pseudoreplicates was inferred by RAxML v.8.2.12 (Stamatakis, 2014).

### 2.5. Phenotypic and physiological characterization

Colony morphology and pigmentation were observed in solid SWOM after 6 days of incubation, and cell features, such as shape and motility, were examined by phase contrast microscopy (Olympus BX41) from a 72-h liquid culture incubated at 25°C with agitation. The optimal growth conditions were determined in the following ranges: temperature, 4–55°C, intervals of 10°C; NaCl, 0–20% (w/v), intervals of 0.5%; and pH, 5.0–10.0, intervals of 1.0. To test pH and salinity ranges, a basal liquid medium supplemented with 0.1% (w/v) of yeast extract was used, while temperature was tested in SWOM. For pH tests, the medium was adjusted to 3.5% (w/v) of NaCl (sea salts, Sigma-Aldrich, MO, USA) and buffered with MES (pH 5.0–6.0), MOPS (pH 6.5–7.0), Tris (pH 7.5–8.5), or CHES (pH 9.0–10.0) at a final concentration of 50 mM. Anaerobic growth was determined by incubation on MA and SWOM agar plates at 25°C for 72 h in the AnaeroGen™ system (Oxoid). Catalase activity was assessed by adding a 3% (w/v) H_2_O_2_ solution to colonies on a solid medium. Oxidase activity was examined using sticks containing tetramethyl-*p*-phenylenediamine (PanReac AppliChem, Darmstadt, Germany). Other biochemical and physiological tests were evaluated following the characterization methods described by Barrow and Feltham (1993). Hydrolysis of Tween 80, gelatin, starch, DNA, casein, and aesculin, indole production from tryptophan, methyl red, and Voges–Proskauer tests, Simmons's citrate utilization, production of H_2_S, urease, nitrate and nitrite reduction, arginine dihydrolase and anaerobic growth with 5% DMSO were tested by supplementing the appropriated medium with 3.5% (w/v) of NaCl. The metabolic profile was evaluated using the microbial ID assay GEN III MicroPlate™ (Biolog), which determines the phenotypic pattern by assimilating a panel of sugars, alcohols, organic acids, and amino acids as a sole source of carbon and energy. As a reference, we used *Roseibaca ekhonensis* CECT 7235^T^. All phenotypic and physiological tests were determined in triplicate.

### 2.6. Chemotaxonomic analysis

Polar lipid analysis was carried out by Leibniz Institute DSMZ-German Collection of Microorganisms and Cell Cultures GmbH (Braunschweig, Germany) services using the deposited strain V10^T^ (=DSM 112951^T^). In brief, polar lipids were extracted using the modified Bligh and Dyer method (Bligh and Dyer, 1959) and separated by two-dimensional silica gel thin-layer chromatography. Total lipids were revealed by spraying molybdate-phosphoric acid with specific reagents to detect defined functional groups.

The cellular fatty acid methyl ester profile of strain V10^T^ was determined at the CECT, Spanish Type Culture Collection (Valencia, Spain), following the protocol recommended by the MIDI Microbial Identification System (Sasser, 1990). The biomass of strain V10^T^ for the fatty acid determination was obtained from a culture on MA after incubation for 72 h at 30°C. The cellular fatty acid content was analyzed by gas chromatography with an Agilent 6850 gas chromatograph and identified according to the TSBA6 method using the Microbial Identification Sherlock software package (MIDI Inc., 2012).

### 2.7. Identification of BGCs, diversity, and evolution

The main objective of this study was to investigate the secondary/specialized metabolites of BGCs to infer the biosynthetic potential of *Roseibaca* sp. V10^T^ and the related members of the family *Paracoccaceae* from an evolutionary perspective. The biosynthetic profiling study involved genome functional annotation using Prokka v.1.14.6 (Seemann, 2014); identification of secondary metabolite gene clusters with antiSMASH v.7.0 (RRID: SCR_022060) (Blin et al., 2023); gene cluster similarity analysis by means of BiG-SCAPE (Biosynthetic Gene Similarity Clustering and Prospecting Engine) (RRID: SCR_022561) v.1.1.2, and CORASON (CORe Analysis of Syntenic Orthologs to prioritize Natural Product Biosynthetic Gene Clusters) (Navarro-Muñoz et al., 2020) tools. The core genes within the BGCs detected by antiSMASH were used to compute cluster sequence similarities by comparison with experimentally characterized genes encoding the biosynthesis of known chemical molecules annotated in the Minimum Information about a Biosynthetic Gene Cluster (MIBiG 3.0) repository (Terlouw et al., 2023). Furthermore, based on sequence similarity networks that encode the biosynthesis of highly similar or identical molecules, BGC sequences from each genome were linked to enzyme phylogenies to create gene cluster families (GCFs). The application of this pipeline allowed us to obtain a global biosynthetic profile to predict the potential of all species to produce novel compounds.

To determine the BGC homologies with known patented proteins, the core sequence within each BGC was translated into amino acids and further searched against non-redundant protein sequence (nr) and patented protein sequence (pataa) databases using BLASTp+ (RRID: SCR_001010). Assigning proteins to families was performed using the Pfam (RRID: SCR_004726) database (Mistry et al., 2021).

## 3. Results

### 3.1. The strain V10^T^ is a novel photosynthetic bacterial species

The initial molecular identification of the strain V10^T^ based on the 16S rRNA prokaryotic marker sequence and subsequent genome-based phylogeny (calculated after concatenation of the translated 595 single-copy core genes) and OGRIs confirmed its taxonomic affiliation. The species characterization was completed with the polyphasic study, which included phenotypic and chemotaxonomic approaches.

According to the almost complete 16S rRNA gene sequence (1432 bp) (NCBI accession number: MW785571) comparison, strain V10^T^ belongs to the genus *Roseibaca*, showing a top sequence identity of 97.8% with *Roseibaca ekhonensis*, the single validly described bacterial name of this genus. This percentage is below the current threshold of 98.65%, which is widely accepted for species delineation (Chun et al., 2018), suggesting that this bacterium might represent a novel taxon within the genus *Roseibaca*.

**Figure 1:**
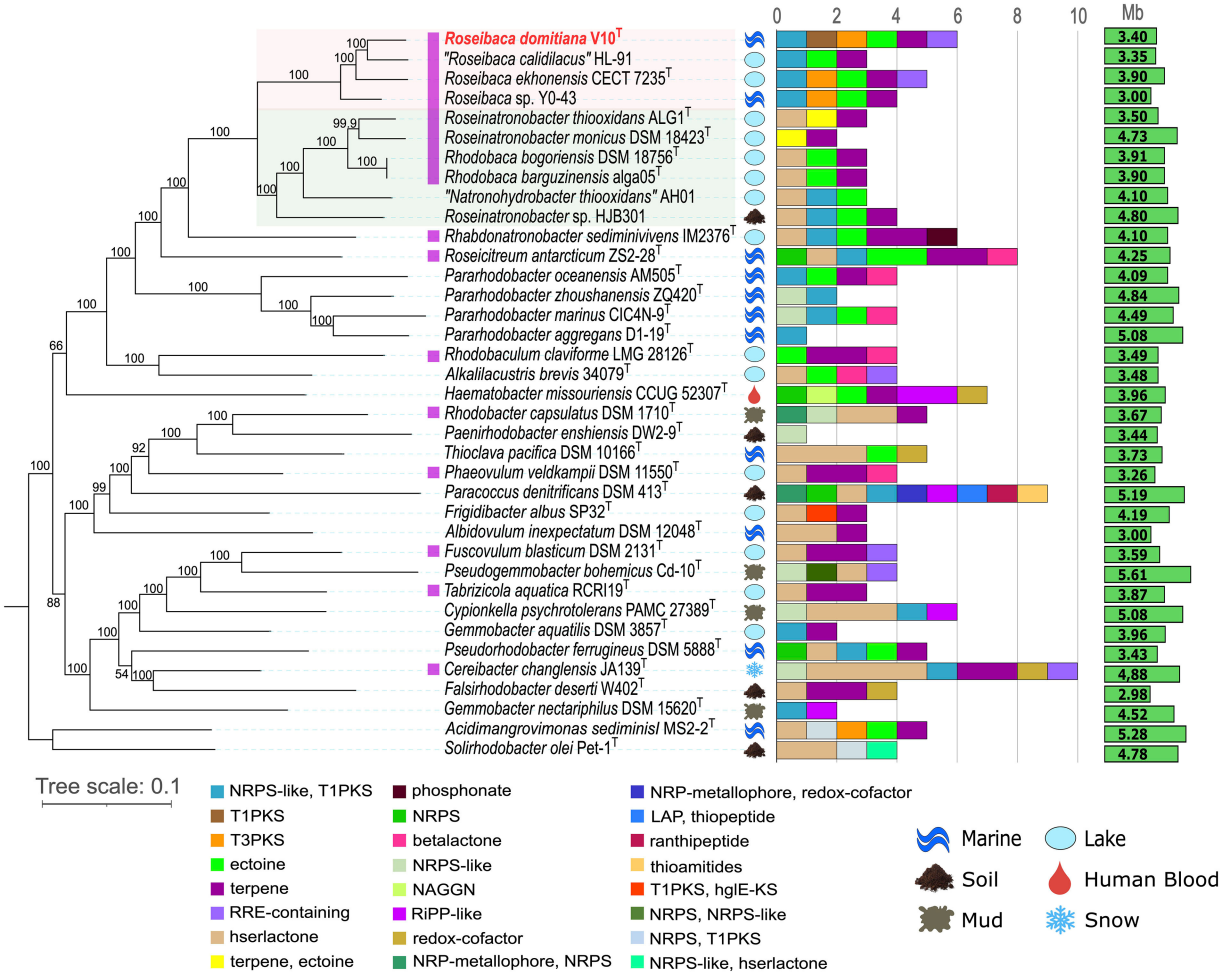
Maximum-likelihood phylogenomic tree coupled with BGC count and diversity showing the relationships between strain *Roseibaca domitiana* V10^T^ and representative species of the most closely related genera. Photosynthetic species are marked in purple. The tree is based on the concatenation of 595 amino acid alignments. Bootstrap percentages are shown next to the branches. Bar, 0.1 substitutions per amino acid position. The isolation source and genome size for each taxon are also displayed. Pink and green shaded areas highlight the other named species of the genus *Roseibaca* and the closest genera within the family *Paracoccaceae*, respectively.

The phylogenetic tree based on the 16S rRNA gene sequences (Supplementary Figure S1) showed that strain V10^T^ is clustered with species of the genus *Roseibaca*, although independently and distantly branched. The other two closest genera were *Rhodobaca* and *Roseinatronobacter*. A rather similar topology was retrieved after the core-genome phylogenomic reconstruction, but with strain V10^T^ clustering together to “*Roseibaca calidilacus*” and *Roseibaca ekhonensis*, while *Roseibaca* sp. Y0-43 falls into a separate branch (Figure 1). This phylogenomic tree is coupled to their BGC profile and discussed in Section 3.2. The clade made up of species of the genus *Roseibaca* includes bacteria derived from marine/saline/hypersaline sources, while the genera *Roseinatronobacter* and *Rhodobaca* harbor alkaliphilic species derived from soda lakes.

Since the species of the genera *Roseibaca, Rhodobaca*, and *Roseinatronobacter* comprise Aerobic Anoxygenic Phototrophic (AAP) bacteria, a phylogenomic reconstruction based on the photosynthetic genes encoding proteins for bacteriochlorophyll production (*pufL, pufM, puhA, bchX, bchY*, and *bchZ*) was inferred. In the tree obtained from the concatenated alignments of these protein-translated photosynthetic genes (Supplementary Figure S2), it was observed that there is a relationship among AAP species, where strain V10^T^ has a similar classification to the previous phylogenetic analyses. Clustered with the species *Roseibaca* and close to the most related species, the strain V10^T^ also constitutes a photosynthetic microorganism.

**Figure 2:**
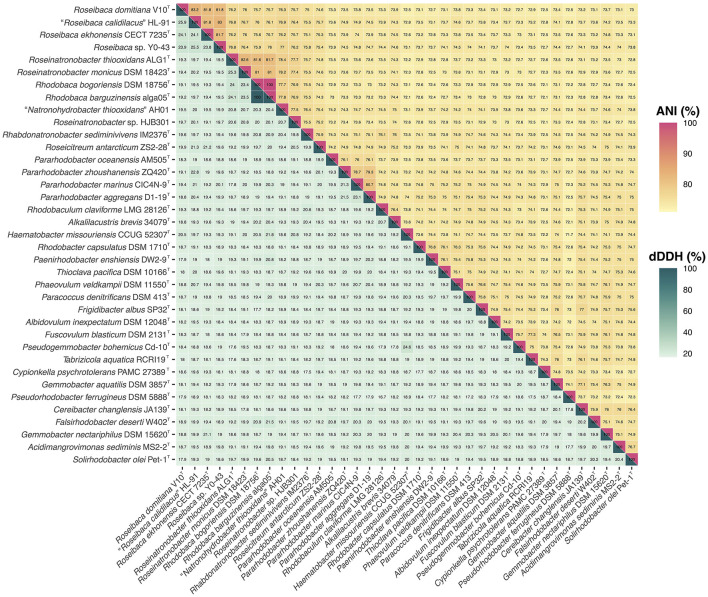
Heatmap of ANI (upper triangle) and dDDH (lower triangle) relatedness among the genome sequences of all the 37 species included in the study.

The OGRI values between *Roseibaca* sp. V10^T^ and the most closely related type strain, *Roseibaca ekhonensis* CECT 7235^T^, were 24.1% for dDDH and 81.8% for ANIb. Both OGRIs are below the current thresholds for species circumscription, that is, 70% dDDH (Auch et al., 2010) and 95–96% ANI (Konstantinidis and Tiedje, 2005; Goris et al., 2007; Richter and Rosselló-Móra, 2009; Chun and Rainey, 2014). Similarly, the OGRI values of *Roseibaca* sp. V10^T^ with respect to the non-type strains of species “*Roseibaca calidilacus*” HL-91 and *Roseibaca* sp. Y0-43 were under these thresholds, as shown in the heatmap (Figure 2).

Overall, single-gene, bacteriochlorophyll multi-locus, and core-genome phylogenies, as well as OGRI results, clearly support that strain *Roseibaca* sp. V10^T^ constitutes a novel species of the genus *Roseibaca*. Additionally, dDDH and ANIb data displayed in Figure 2 point to taxonomic inconsistencies within the family *Paracoccoceae*, in particular the two species of the genus *Rhodobaca, R. barguzinensis* and *R. bogorensis*, both exceeding the species cutoff boundaries with a 100% identity. According to these indices and the very close relationship between them observed in the phylogenomic study, these strains are members of the same species as previously reported (Liang et al., 2021); thus, we confirm that these species names are synonyms.

Phenotypically, *Roseibaca* sp. V10^T^ forms pale to pink-pigmented colonies after 4 days of aerobic and light-dark cycle incubation on natural SWOM at ~25°C. Pigments are not produced when the strain grows in the absence of vitamin solution (B complex) and limiting light exposure; thus, this explains why this bacterium can display a fading pigmentation from pink to pale. Physiologically, strain V10^T^ is halophilic and alkali-tolerant, not being able to grow with <3% (w/v) NaCl and outside the pH range of 6.0 to 9.0. Cells are Gram-stain-negative rods with a size ranging from 1.5 to 3.0 μm in length and 0.5 μm in width, presenting gliding motility with a tendency to occur in long irregular chains, showing a budding process and exhibiting globular shapes (Supplementary Figure S3).

The metabolic profile evaluated by means of the Biolog phenotypic system (Supplementary Figure S4) showed that *Roseibaca* sp. V10^T^ has low nutritional requirements, using few carbon and energy sources. The chemotaxonomic study revealed that the polar lipid pattern (Supplementary Figure S5) consists of phosphatidylglycerol as a major polar lipid, diphosphatidylglycerol, an aminolipid, a phospholipid, and an unidentified lipid. In contrast to *R. ekhonensis*, phosphatidylethanolamine, and phosphatidylcholine were absent in *Roseibaca* sp. V10^T^. The cellular fatty acid composition comprises C_18:1_ ω7c/C_18:1_ ω6c as the major (>79.7%) phospholipid fatty acid (PLFA), followed by minor fatty acids (< 8%), namely C_16:1_ ω7c/C_16:1_ ω6c and C_19:1_ ω6c/C_19:0_ cyclo. Further physiological and chemotaxonomic data are detailed in the species description and the differential table with respect to the most closely related type species, *Roseibaca ekhonensis* (Table 1).

**Table 1:** Differential features between *Roseibaca* sp. V10^T^ and *Roseibaca ekhonensis* CECT 7235^T^.

**Feature**	***Roseibaca* sp. V10^T^**	***Roseibaca ekhonensis* CECT 7235^T^**
Cell size [width × length; μm]	0.5 × 1.5−3.0	1.0 × 2.5
NaCl (%, w/v) growth range	3−10%	0−4%
Temperature range	10 − 30 °C	10 − 30 °C
Motility (gliding)	+	−
Colony pigmentation	Pink pale/pink	Pink
pH range	6.0 − 9.0	7.0 − 9.5
**Utilization of:**		
D-maltose	−	+
D-fructose 6-PO_4_	+	−
Gentiobiose	+	−
D-melibiose	+	−
Tween 40	−	+
D-galacturonic acid	−	+
D-glucoronic acid	−	+
β-hydroxy-D, L-butyric acid	+	−
L-lactic acid	+	−
Citric acid	−	+
N-acetyl D-galactosamine	−	+
N-acetyl-D-glucosamine	−	+
**Chemotaxonomy**		
Phosphatidylethanolamine (PE)	−	+ ^*^
Phosphatidylcholine (PC)	−	+ ^*^
Major fatty acid	C_18:1_ ω7c/C_18:1_ ω6c	C_18:1_ ω7c ^*^
DNA G+C [mol%, genome]	60.5	61.6

All data from this study unless otherwise indicated. +, positive reaction; −, negative reaction.

* Data taken from the author's description (Labrenz et al., 2009).

Based on the phylogenetic/phylogenomic reconstructions and the genomic comparisons, together with the physiological features, we can conclude that the strain V10^T^ constitutes a novel species within the genus *Roseibaca*, for which the name *Roseibaca domitiana* sp. nov. is proposed. The type strain is V10^T^, and it is publicly available in the following culture collections: Spanish Type Culture Collection (CECT) as CECT 30319^T^; German Collection of Microorganisms and Cell Cultures GmbH (DSMZ) as DSM 112951^T^; and Laboratory of Microbiology of Ghent University/Belgian Coordinated Collections of Microorganisms (LMG/BCCM) as LMG 32429^T^.

#### 3.1.1. Description of *Roseibaca domitiana* sp. nov.

*Roseibaca domitiana* (do.mi.ti.a'na. L. fem. adj. *domitiana*, belonging to the Domitian littoral).

Cells are Gram-stain-negative rods, measuring 1.5–3.0 × 0.5 μm in size. Colonies are pale to pink-pigmented, 2 mm in diameter, with a cream consistency and smooth aspect on seawater oligotrophic medium (SWOM) after 4 days of incubation at 25°C. Similar features are observed in marine agar. The pink pigment is reduced when growth occurs in the absence of vitamins and light. The bacterium shows gliding motility and forms long irregular chains exhibiting a budding process with globular shapes in 4-day-old cultures: aerobic anoxygenic phototrophic (AAP), bacteriochlorophyll *a*-producing, and facultative photoheterotrophic bacterium.

Halophilic and alkali-tolerant, it is able to grow in a range from 3 to 10% (w/v) NaCl at 10 to 30°C and pH 6.0 to 9.0, with optimal growth at 3.5% (w/v) NaCl at 25°C and pH 7.5. Catalase and oxidase are negative. Nitrate is reduced, but nitrite is not. Tween 80, starch, DNA, aesculin, urea, casein, and gelatin are not hydrolyzed. Indole and H_2_S are not produced. Voges-Proskauer and methyl red tests yield negative results. The organism utilizes various compounds including D-fructose 6-PO_4_, D-glucose 6-PO_4_, L-glutamic acid, L-histidine, glucuronamide, L-lactic acid, α-keto-glutaric acid, L-malic acid, α-hydroxy-butyric acid, β-hydroxy-D, L-butyric acid, α-keto-butyric acid, and acetoacetic acid as its main sources of carbon and energy. It demonstrates the ability to grow in the presence of sodium butyrate, 5% DMSO, and the following antibiotics: troleandomycin, rifamycin, minocycline, lincomycin, vancomycin, nalidixic acid, and aztreonam. The polar lipid profile consists of phosphatidylglycerol as a major lipid, diphosphatidylglycerol, an aminolipid, a phospholipid, and an unidentified lipid. The cellular fatty acid composition comprises C_18:1_ ω7c/C_18:1_ ω6c as the major fatty acid (>79.7%). The DNA G+C content is 60.5 mol% (genome).

The type strain is V10^T^ (= CECT 30319^T^ = DSM 112951^T^ = LMG 32429^T^), isolated from seawater in the Domitian littoral, Italy. The GenBank/EMBL/DDBJ accession numbers for the 16S rRNA gene and the whole-genome sequences of the type strain are MW785571 and JALZWP000000000, respectively.

### 3.2. Biosynthetic gene profiling and genomic potential

#### 3.2.1. Phylogenetic distance is uncorrelated with the BGC profile

The theoretical biosynthetic profile of *Roseibaca domitiana* and related strains was determined by assessing the secondary/specialized metabolism through BGC predictions. Then, the identified BGCs were coupled to the whole-genome-based phylogeny, which allowed us to establish the relationships between phylogenetic distance and BGC diversity.

The BGC predictions obtained by the antiSMASH tool revealed that the species of the family *Paracoccoceae* investigated in this study (those closely related to *Roseibaca domitiana*) harbored a low BGC count, ranging from one to 10 BGC regions per genome and spanning a total of 24 different types of clusters (Figure 1). The studied strains belonging to different genera within the *Paracoccoceae* came from different environmental sources, especially from marine and aquatic extreme habitats, such as alkaline and hypersaline lakes. The phylogenomic tree, coupled with the BGC count, showed that each species possesses a different BGC profile, except for the genus *Rhodobaca*, which shared the same profile. In all cases, the observed patterns were independent of both the isolation source and the genome size. The global profile also displayed a predominance of some BGC types, such as homoserine lactone (hserlactone) and terpene; this latter was often identified in photosynthetic species that produce bacteriochlorophyll (marked in purple), and it is linked to the ability to synthesize carotenoid pigments, one of the main features of the purple bacteria here studied. Less abundant types of BGCs were the hybrid NRPS/T1PKS and ectoine, both distributed across species of different genera, though the latter was identified in some species of aquatic, soil, or clinical origin, such as *Hematobacter missouriensis*. The presence of NRPS/T1PKS is linked to the ability to synthesize bioactive molecules, while ectoine is related to the osmoprotection to survive in saline environments, as in the case of most of the species in the study.

It is important to note that most of the BGCs belonging to the categories PKS and NRPS are present as hybrid clusters. This means that the cluster is composed of more than one type of BGC located or overlapped in the same region of the genome, and, therefore, hybrids were classified into different categories according to the combination of BGC types they were made of.

Focusing on the genus *Roseibaca*, all its four cultivated members possessed a common biosynthetic profile consisting of three shared BGCs: NRPS-like/T1PKS hybrid, ectoine, and terpene. With the exception of “*R. calidilacus*,” T3PKS was also present in species of this genus. Additionally, only *R. domitiana* and *R. ekhonensis* shared the RRE-containing cluster. Remarkably, the new species proposed here, *R. domitiana*, and the only validly named one, *R. ekhonensis*, had a very similar biosynthetic pattern despite having been isolated from different environments, the former from a marine habitat and the latter from a hypersaline lake. On the other hand, the two validly named species of *Rhodobaca* shared the same profile. However, this was not unexpected considering that both species are synonyms in previously reported studies and in our comparative genomic results. Regarding the relationship between BGC content and genome size, no direct association was observed since one of the largest genomes analyzed, i.e., *Pararhodobacter aggregans* D1-19^T^ (5.08 Mb), exhibited only one BGC, while some of the smallest (< 4.0 Mb), i.e., *Roseibaca domitiana* V10^T^ harbored six BGCs.

Overall, most of the studied species displayed a differential BGC profile that was not related to the phylogenetic distance among the taxa. In addition, it was found that the source of isolation and the size of the genome are not linked to the biosynthetic profile of the species within the family *Paracoccoceae*. Only a resolute BGC pattern that allowed us to distinguish between the photosynthetic genera *Roseibaca, Roseinatronobacter*, and *Rhodobaca* could be established. This observation will be strengthened when more genomes of species in these genera become available.

#### 3.2.2. The vast majority of BGCs are involved in the synthesis of unknown molecules

The biosynthetic potential of *Roseibaca domitiana* and its closely related species was determined based on the novelty of the predicted BGCs in each genome, based on the homology with characterized genes encoding the synthesis of known chemical molecules present in the MIBiG repository and verified *in vitro*.

**Figure 3:**
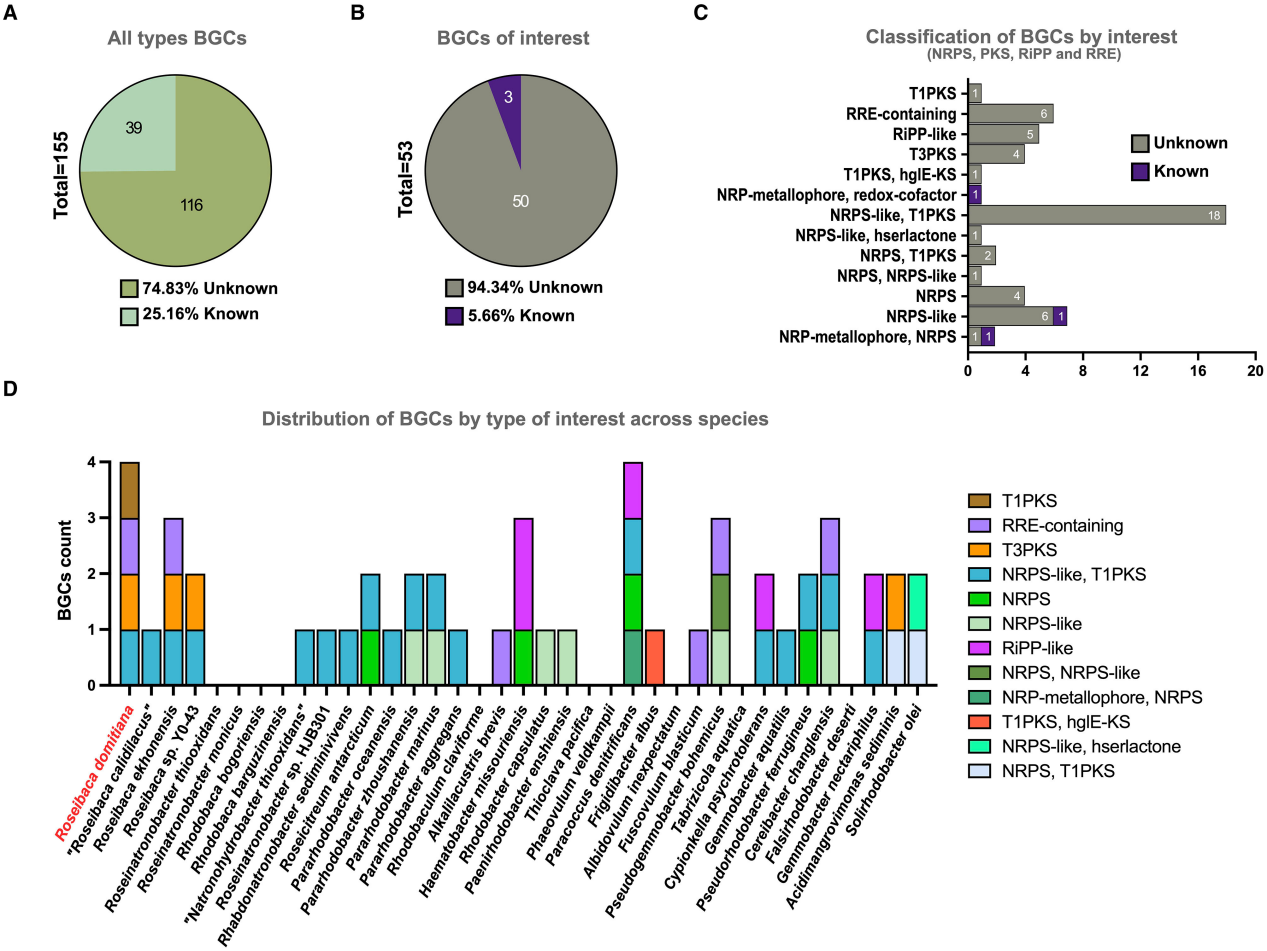
Total BGC count by categories across the 37 analyzed genome sequences. **(A)** Percentage of known vs. unknown BGCs, considering all types. **(B)** Percentage of known vs. unknown BGCs, considering only types with biotechnological interest. **(C)** BGC counts by categories of biotechnological interest. **(D)** BGC counts by categories of biotechnological interest distributed across the studied species.

A total of 155 BGCs assigned to 24 different categories were predicted in the 37 analyzed genomes, as aforementioned. A total of 74.83% of the detected BGCs are unknown (Figure 3A) since no similarities were found with any known cluster involved in the synthesis of chemical entities. Focusing on the BGC types of biotechnological interest (i.e., NRPS, PKS, RiPP, and RRE), 53 BGCs were identified across the studied genomes, 94.34% of which were completely unknown (Figures 3B, C). These promising novel BGCs were distributed among 27 out of the 37 analyzed species, which may represent potential producers of new molecules (Figure 3D). Concerning the type of these new BGCs, the most predominant was the hybrid NRPS-like/T1PKS since it was detected in 18 species. Furthermore, this hybrid BGC was found in all the species of *Roseibaca*, so far representing a common feature of the members of this genus.

Regarding *Roseibaca domitiana*, a total of six BGCs were predicted (terpene, T3PKS, NRPS-like/T1PKS, ectoine, RRE-containing, and T1PKS), of which only terpene and ectoine showed high levels of sequence similarity (100 and 80%, respectively) with known clusters that encode the synthesis of carotenoids (squalene/phytoene) and ectoine, respectively. On the contrary, T3PKS, RRE-containing, and T1PKS did not match with any known cluster or had a low similarity (>8%) with known BGCs present in the MIBiG database, as in the case of the hybrid NRPS-like/T1PKS (Supplementary Table 2). It is important to consider the relevance of T1PKS since it is the unique BGC that distinguishes *Roseibaca domitiana* with respect to other species of the genus.

#### 3.2.3. Novel BGCs of *Roseibaca domitiana* are conserved only at the genus level

As has been previously indicated, *Roseibaca domitiana* V10^T^ possesses six BGC types, of which four are considered new: NRPS-like/T1PKS, T1PKS, T3PKS, and RRE-containing. Here, we determine the similarity/diversity of these BGCs and the evolutionary relationships among the others predicted across the studied species to evaluate their biosynthetic potential.

Their diversity was evaluated by classifying them into GCFs based on sequence similarity networks and further linking them to enzyme phylogenies. Likewise, the evolutionary relationships among these BGCs were inferred through their core sequence and additional multi-locus phylogenies that allowed us to identify the BGC conservancy in *Roseibaca domitiana* V10^T^ with respect to those of other species, therefore establishing their biosynthetic potential.

**Figure 4:**
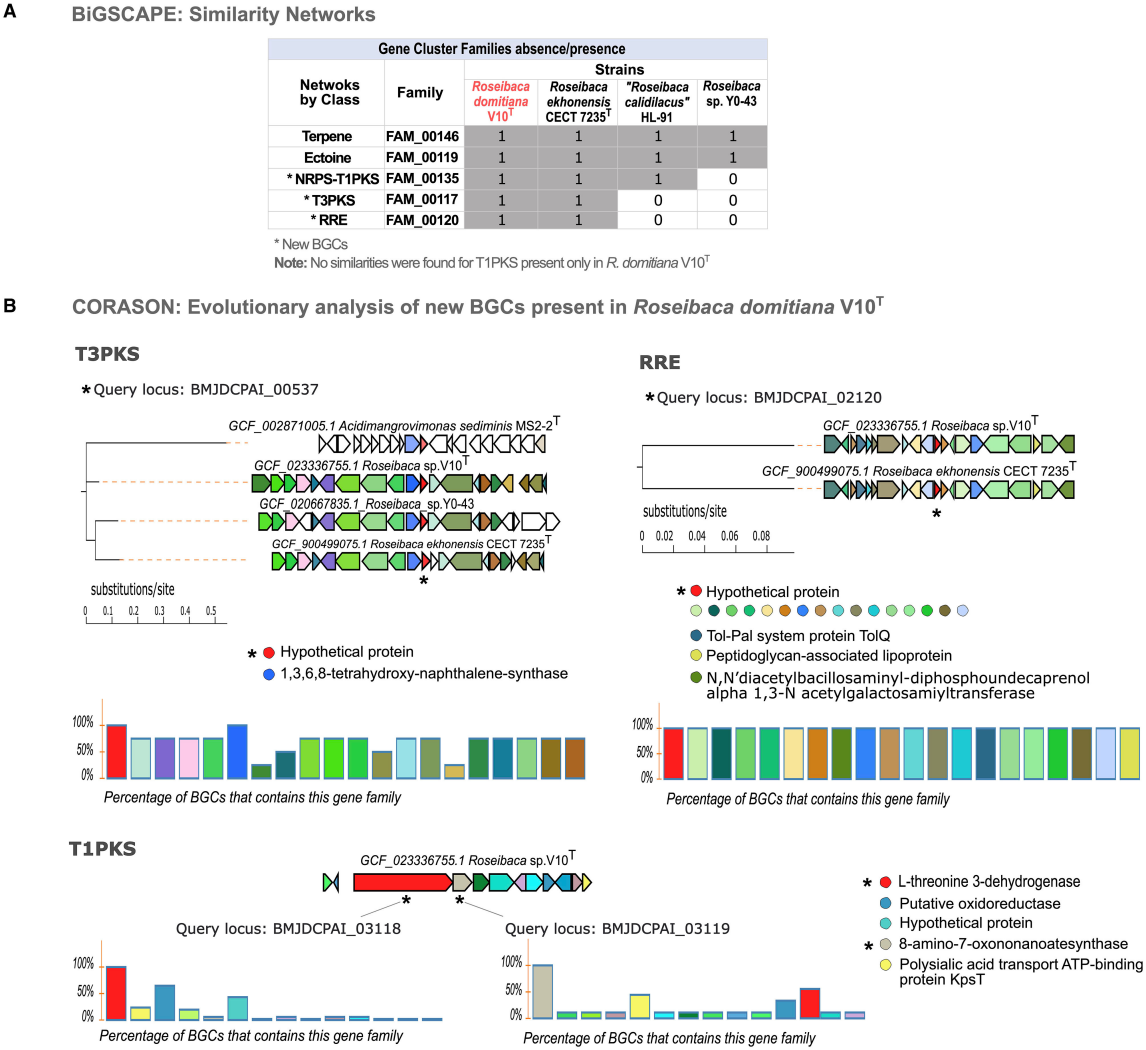
Similarity networking and evolutionary analysis of BGCs. **(A)** Absence/presence matrix of GCFs across species of the genus *Roseibaca*. **(B)** Multi-locus phylogeny based on the core sequence of the three new BGCs across studied species, (the query is specified with an asterisk). The charts show the level of synteny between species when an evolutionary relationship is found. In colored circles, the domains that cover 100% (highly conserved). In the case of T1PKS, those less conserved domains are also shown, highlighting the uniqueness of this BGC. T1PKS is displayed as a singleton (evolutionarily unrelated to other species).

The similarity network analysis carried out with BiGSCAPE showed that the BGCs from *Roseibaca* form five GCFs of a different class (Figure 4A): FAM_00146 and FAM_00119, made up of all species of this genus and belonging to terpene and ectoine, respectively; FAM_00135, made of three species excluding *Roseibaca* sp. Y0-43 belonging to the NRPS-T1PKS hybrid class; and finally, FAM_00117 and FAM_00120, both made of *R. domitiana* V10^T^ and *R. ekhonensis* CECT 7235^T^ belonging to T3PKS and RRE categories, respectively.

Since the networking is based on the similarity of BGCs, the families here obtained are not made of the species that contain the same type of cluster, as could be expected in the case of the hybrid NRPS-T1PKS predicted in all strains of *Roseibaca*, and T3PKS predicted also in *Roseibaca* sp. Y0-43 but not classified within the same family. It is important to note that no similarities were found for T1PKS of *R. domitiana* V10^T^ among the studied species; therefore, it remains unclassified without forming a GCF. As displayed in the GCF absence/presence matrix (Figure 4A), *R. domitiana* V10^T^ only shared core enzyme-coding genes with species of its own genus, and, in the case of the unclassified BGC T1PKS, this remains exclusive of *R. domitiana* V10^T^.

Once homologous BGCs based on protein domain content and sequence identity were identified, we addressed the biosynthetic potential from an evolutionary perspective by using CORASON software. Through this tool, a phylogenetic reconstruction of BGCs was obtained based on syntenic orthologs in the BGC core shared between species (Figure 4B). In this way, the phylogenies obtained from the four novel BGCs (NRPS-T1PKS, T3PKS, RRE, and T1PKS) predicted in *R. domitiana* V10^T^ showed that the hybrid NRPS-T1PKS was present and conserved in all species of *Roseibaca*, but non-confined to this genus, as it is partially shared with some species from other genera (Supplementary Figure S6). As observed in the phylogenetic reconstruction of T3PKS, this cluster is present and highly conserved in *R. domitiana* V10^T^*, R. ekhonensis*, and *Roseibaca* sp. Y0-43, which exhibits a high synteny and shares more than 60% of this BGC. Similar is the case with RRE, which is present only in the genomes of *R. domitiana* V10^T^ and *R. ekhonensis* and shares 100% of the genes that conform to this cluster, which means that this BGC is fully conserved in both species. Regarding T1PKS (predicted only in *R. domitiana* V10^T^), the phylogenetic reconstruction of this BGC was based on two genes (core and additional ones) as queries to investigate its presence and conservation in the genomes of the species under study. Both genes confirmed that T1PKS is unique to *Roseibaca domitiana* V10^T^, since this cluster is present in other species with low or no homology, which indicates that this BGC may be present in multiple genomes, but the genes involved in the cluster are not conserved in these genomes (Supplementary Figure S7).

Finally, to reveal if these BGCs from *R. domitiana* codify enzymes matching already available commercial products or patented proteins, their core amino acidic sequence was BLASTp+ searched against the database of patented proteins. The results indicated that the novel BGC-derived proteins only matched < 58% similarity with active patents (Table 2). In detail, T3PKS and T1PKS are remotely related to patents with plant growth promotion applications, while NRPS-T1PKS and RRE are related to the antitumoral argyrin synthetic pathway enzymes, indicating that *R. domitiana* has the potential to synthesize new molecules, possibly with similar functions.

**Table 2:** Amino acid sequence identity of novel BGCs from *Roseibaca domitiana* V10^T^ searched against a patent protein database.

**Region/Type**	**Query locus tag/encoded protein**		**Location (size)**	**Patent hit/similarity**	**Application (status)/reference**
2.1 T3PKS	BMJDCPAI_00536 1,3,6,8-tetrahydroxynaphthalene synthase		124,396 - 125,436 (1,041 nt)	US 7314974 57.40%	Expression of microbial proteins in plants for the production of plants with improved properties (active) (Cao et al., 2008)
2.2 NRPS-T1PKS	NRPS	BMJDCPAI_00765 D-alanine–D-alanyl carrier protein ligase	350,790 - 355,316 (4,527 nt)	US 6833447 41.03%	EP 2297181 B1 Synthetic pathway enzymes for the production of argyrins (active) (Müller et al., 2013)
	T1PKS	BMJDCPAI_00767 hypothetical protein	356,309 - 362,641 (6,333 nt)	US 6833447 44.87%	
9.1 RRE-containing	BMJDCPAI_02120 hypothetical protein		106,516 - 106,791 (276 nt)	US 6833447 29.23%	
26.1 T1PKS	BMJDCPAI_03118 L-threonine 3-dehydrogenase		2,994 - 10,304 (7,311 nt)	US 8343764 36.74%	Genes encoding glutamine synthetase and use for plant improvement (active) (Abad et al., 2013)

## 4. Discussion

Marine microbiota is considered among the most promising natural resources for new drug discovery in biomedical and biotechnological fields. However, a large proportion of the microbial diversity remains hidden in terms of cultivation. Hence, the main challenge is indeed uncovering and isolating this “microbial dark matter”. Once this goal is achieved, what is sought is not only to understand the natural environment to improve and simplify access to microbial resources but also to perform a deep and effective study of the physiology, ecological role, and possible applications of these novel isolated microorganisms. The biotechnological potential of new strains requires a comprehensive evaluation of their biosynthetic abilities before undertaking efforts that may entail the rediscovery of already known natural products, a frequent issue in drug discovery projects.

The results obtained here are derived from seasonal and diel samplings conducted in the Tyrrehenian Sea in Italy. This strategy was employed to isolate new microbial species that belong to the as-yet uncultured marine microbiota. In this way, we achieved the isolation of strain V10^T^, initially identified as a member of *Roseibaca*, a little-known genus represented by a single species, *Roseibaca ekhonensis*, recovered from a thermal hypersaline lake in Antarctica (Labrenz et al., 2009). Our phylogenomic and polyphasic studies have revealed that strain V10^T^ represents a new species within this genus; we propose naming it *Roseibaca domitiana* sp. nov. Given that this strain was isolated from winter nocturnal samples of the coastal surface, while *R. ekhonensis* was cultured from the depths of a hypersaline lake, the isolation of strain V10^T^ supports the use of night-time and seasonal sampling as a strategy for the cultivation of new microorganisms, an approach that we successfully used in a previous study and allowed the isolation of *Leeuwenhoekiella parthenopeia*, a new rare biosphere bacterium that inhibits the proliferation of tumor cells (Gattoni et al., 2023).

In addition to the only recognized species of *Roseibaca* (*R. ekhonensis*), our study also included uncharacterized cultivated strains reported as “*Roseibaca calidilacus*” HL-91 isolated from a microbial mat of a hypersaline thermal lake (Romine et al., 2017) and *Roseibaca* sp. Y0-43 of marine origin (unpublished). Considering that these cultivated species come from extreme aquatic environments, *Roseibaca domitiana* represents the only marine species characterized so far. Thus, the genus *Roseibaca* groups marine and polyextremophile (halophilic, thermophilic, and alkali-tolerant) microorganisms and, as such, inhabits marine, saline, hypersaline, and thermal aquatic systems.

The metabolic versatility of *R. domitiana* V10^T^ is striking since it is an aerobic anoxygenic phototrophic bacterium that could also grow in the absence of light and vitamin B complex, but not without NaCl. One of its most remarkably phenotypic features is the potential to synthesize the carotenoid pigment bacteriochlorophyll due to the presence of the *pufL, pufM*, and *puhA* genes, encoding, respectively, the L, M, and H subunits of the photosynthetic reaction center proteins, which enable aerobic anoxygenic photoheterotrophy (Pradella et al., 2004; Zheng et al., 2011). The production of the carotenoid pigment is conditioned by light exposure, with pink (with light) and pale (without light) colonial phenotypes. We also found the *pufLM* and *puhA* genes in the genome sequences of the phylogenetically closest species, such as those of the genera *Roseibaca, Roseinatronobacter*, and *Rhodocaca*, whose light-related physiology might be similar to that of strain V10^T^.

*R. domitiana* is a prokaryote that can grow in a wide range of saline and alkaline conditions, although it utilizes a restricted number of substrates. It belongs to the purple nonsulfur bacteria (PNSB), and its light behavior is compatible with this group of microorganisms, known as nature's preeminent photoheterotrophs, capable of photoautotrophy and with diverse capacities for dark metabolism and growth (Madigan and Jung, 2009). We may hypothesize that its main role in the marine environment would be contributing to the recycling of organic matter and the carbon cycle (Koblížek, 2015; Piwosz et al., 2022).

The biosynthetic gene profiling of the specialized metabolism of *Roseibaca domitiana* and its closest relatives revealed that there is a typical BGC pattern shared among the members of the genera *Roseibaca, Roseinatronobacter*, and *Rhodobaca*, consisting of two BGC types, ectoin, and terpene. The first is related to the production of ectoin, a compatible solute that acts as an osmoprotectant to counteract salt stress (Roessler and Muller, 2001), and the second is involved in the synthesis of carotenoids produced by photosynthetic bacteria (Higuchi-Takeuchi and Numata, 2019). The presence of both of these types of BGCs concurs with their physiology to thrive in the environment where they inhabit.

Since BGC occurrence and distribution are homogenous in these three genera, *Roseibaca, Roseinatronobacter*, and *Rhodobaca*, only in these cases may the BGC allow differentiation at the genus level. A similar finding has also been reported for the species of the actinobacterial genus *Rhodococcus* (Undabarrena et al., 2021). Conversely, the other analyzed genera within the family *Paracoccaceae* showed heterogeneous BGC profiles unrelated to the phylogenetic distance between them, meaning that each species possesses its own diversity of BGC and could synthesize very different molecules. This non-conserved pattern has been previously found for the species of the genus *Streptomyces*, the most prolific source of natural products (Belknap et al., 2020; Alam et al., 2023), and other actinobacteria, such as *Saccharomonospora* (Ramírez-Durán et al., 2021).

Regarding the novelty of the BGCs of biotechnological interest (NRPS, PKS, RRE, and RiPP) of all the studied species, the vast majority possess an uncovered biosynthetic potential since 94.34% of the identified BGCs encode genes involved in the synthetic pathways of chemically unknown compounds. Besides, the most predominant BGC categories found were hybrid NRPS-like and T1PKS, which means that most species could be potential synthesizers of antibiotic and antitumor peptides (Frattaruolo et al., 2017; Nesic et al., 2022).

With respect to *R. domitiana*, it harbored six BGCs unrelated or fairly distant to those of the MIBiG database that could, therefore, be considered novel BGCs. The evolutionary analysis of these new BGCs allowed us to identify two of them (RRE-containing and T3PKS) as highly conserved in *R. domitiana* and *R. ekhonensis*, while another one, i.e., T1PKS, was exclusive to *R. domitiana* and is not related to any known BGC. The latter can be considered “new among the new ones." The amino acid sequence derived from these three BGCs showed an identity of less than 58% with active patented proteins. Specifically, the hybrid NRPS-T1PKS and RRE-containing types displayed very low similarities with enzymes from a European patent involved in the synthetic pathways for the production of argyrins, a potent antitumor under study (Sasse et al., 2002; Müller et al., 2013). Likewise, the T3PKS type was remotely related to a patent involved in plant production improvement in agricultural crops (Cao et al., 2008). The exclusive BGC, T1PKS of *R. domitiana*, was related to genes encoding a glutamine synthetase patented to improve plant production (Abad et al., 2013).

In summary, our study permitted us to conclude that the seasonal and diel sampling approaches increase the probability of isolating new species with not-yet-described biochemical and physiological features. Besides, the exploration of secondary/specialized metabolism combined with phylogenomic and evolutionary studies is shown to be a valuable strategy to assess the biosynthetic potential of new microbial groups, either at the species level or in an overall context. Finally, this and other recent studies (Ramírez-Durán et al., 2021; Gattoni et al., 2023) show that rare and minority bacteria harbor untapped metabolic potential for synthesizing novel, biotechnologically significant compounds. A good example is the new marine bacterium *R. domitiana* V10^T^, which is theoretically able to produce plant-promoting compounds and antitumor molecules, which merits prioritizing future *in vitro* studies. The research conducted here encourages the exploitation of microbial minorities as promising, realistic sources for new compounds in agricultural and pharmaceutical applications.

## Data availability statement

The datasets presented in this study can be found in online repositories. The names of the repository/repositories and accession number(s) can be found in the article/Supplementary material.

## Author contributions

GG and PC: investigation, formal analysis, and writing of the original draft manuscript. GG, FDC, RRH, ABF, SG-F, NS-M, and PC: methodology, software, and visualization. GG and FDC: data curation. RRH, NS-M, AV, and PC: validation. NS-M, AV, and PC: funding acquisition, project administration, and resources. PC: conceptualization and supervision. All authors contributed to the manuscript revision, read, and approved the submitted version.
