# Supplementary material for: Biosynthetic gene profiling and genomic potential of the novel photosynthetic marine bacterium Roseibaca domitiana
Source: Front Microbiol. 2023 Sep 29;14:1238779. doi: 10.3389/fmicb.2023.1238779 (PMC10584327; doi:10.3389/fmicb.2023.1238779)
Supplement: Supplementary file 1 [file Data_Sheet_1.PDF]

Supplementary Material

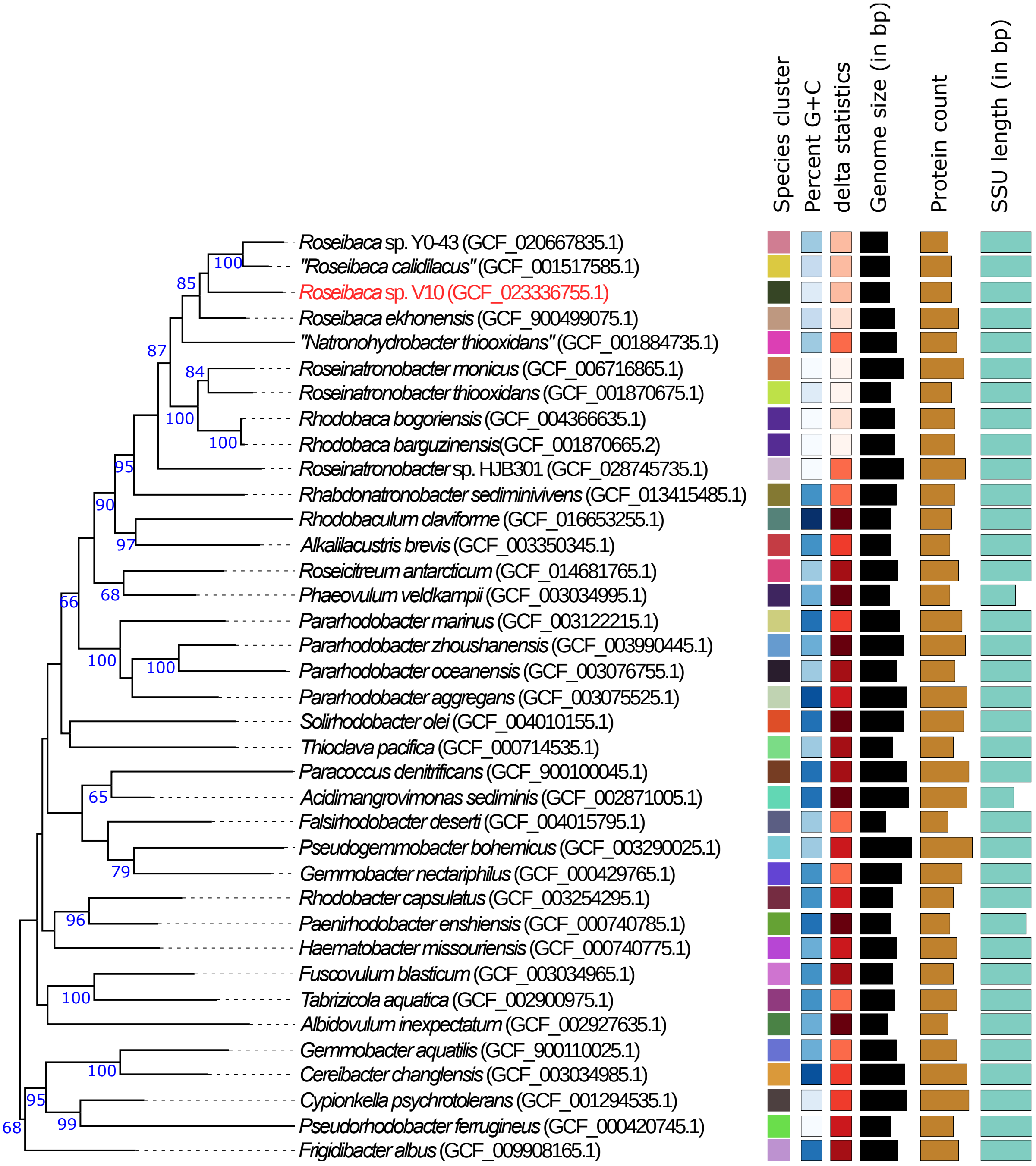

Supplementary Figure S1. Tree inferred with FastME 2.1.6.1 from GBDP distances calculated from 16S rDNA gene sequences. The branch lengths are scaled in terms of GBDP distance formula d_5. The numbers above branches are GBDP pseudo-bootstrap support values > 60 % from 100 replications, with an average branch support of 68.8 %. The tree was rooted at the midpoint.

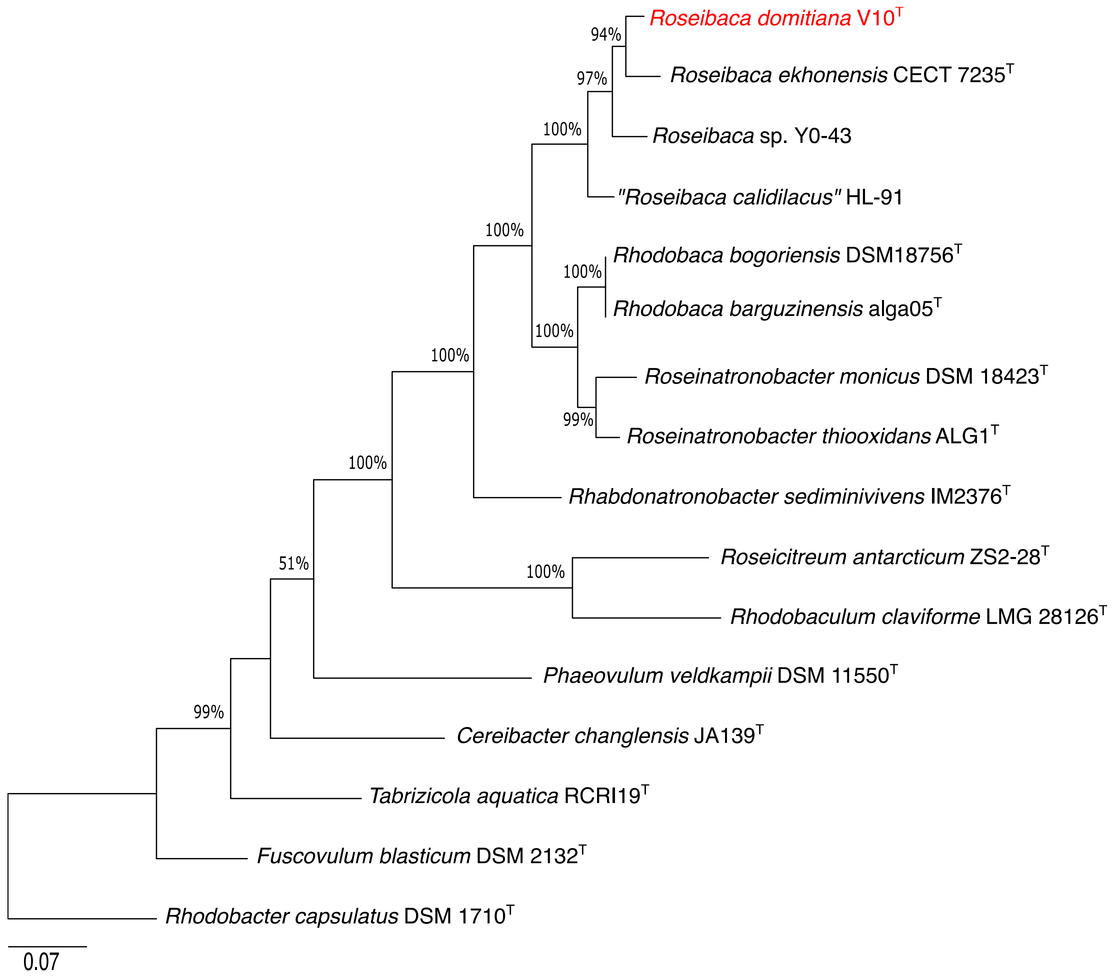

Supplementary Figure S2. Phylogenetic analysis of *pufLM*, *puha*, and *bchXYZ* concatenated protein sequences. Maximum-likelihood tree was constructed based on a WAG amino acid substitution model. Numbers next to the tree branches indicate bootstraps (1000 replicates).

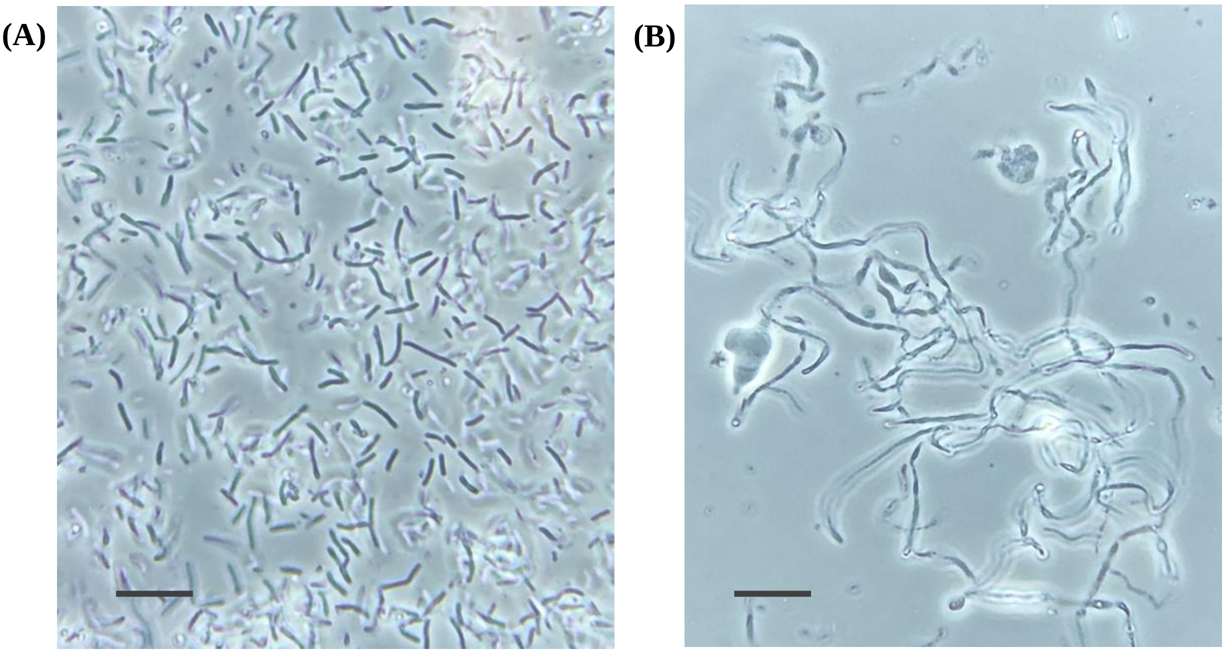

Supplementary Figure S3. Phase-contrast microphotographs of *Roseibaca domitiana* strain V10^{T}. (A) Image after 72 hours of incubation (exponential phase) with some cells exhibiting budding process. (B) Image after 10 days of incubation showing long chains and globular shapes. Scale bar, 10μm.

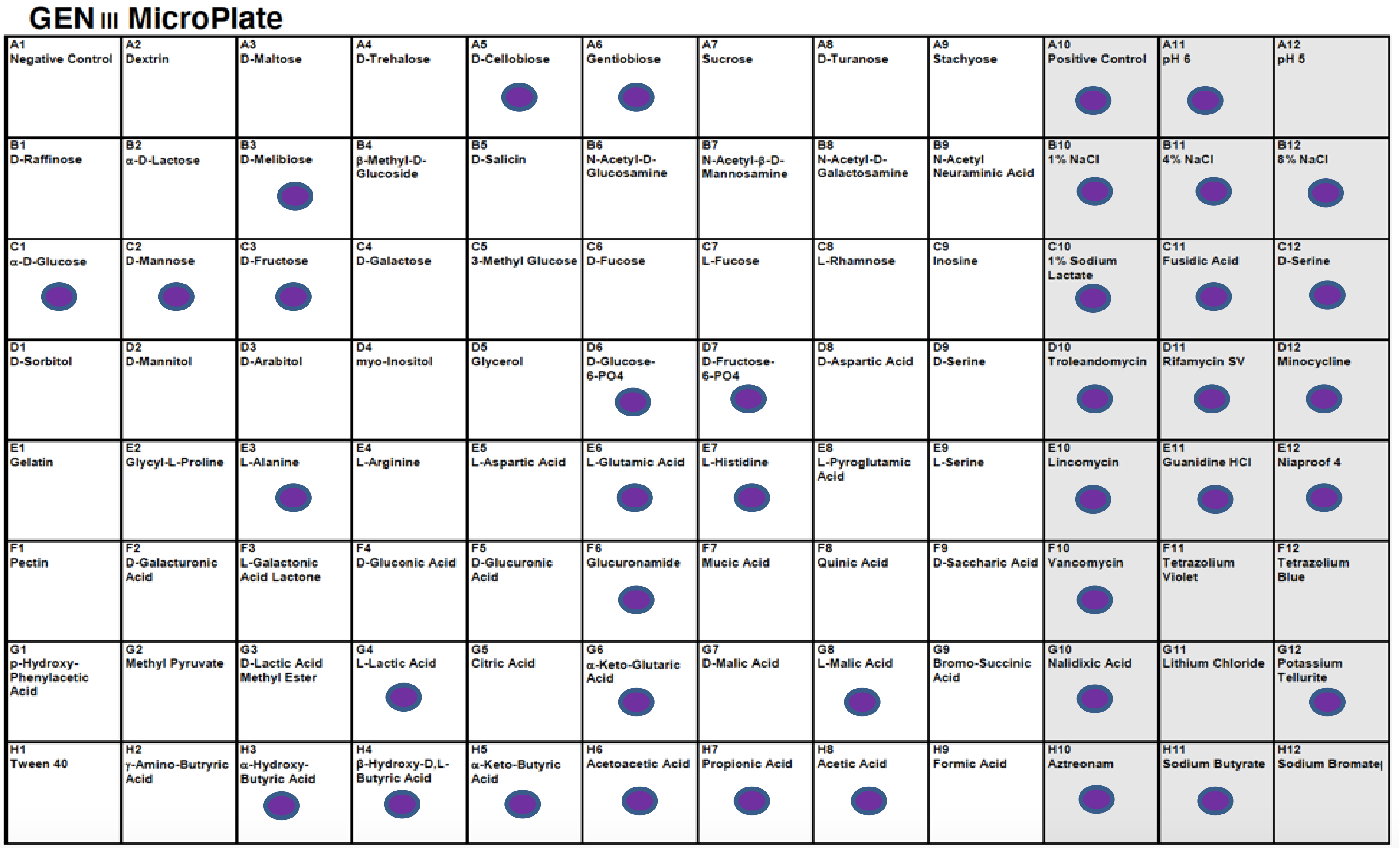

Supplementary Figure S4. Biolog GEN III MicroPlate test panel showing physiological and metabolic profile of *Roseibaca domitiana* V10^{T}.

DPG = Diphosphatidylglycerol

PG = Phosphatidylglycerol

AL = Aminolipid

PL = Phospholipid

L = Lipid

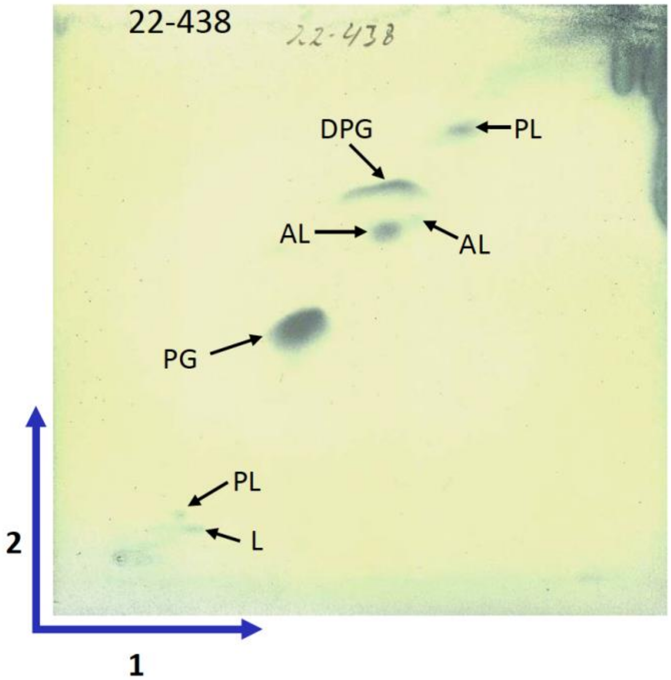

Supplementary Figure S5. Two-dimensional thin layer chromatography showing the polar lipid composition of *Roseibaca domitiana* V10^{T} from deposited material (DSM 112951^{T}). The plate was revealed with phosphomolybdic acid.

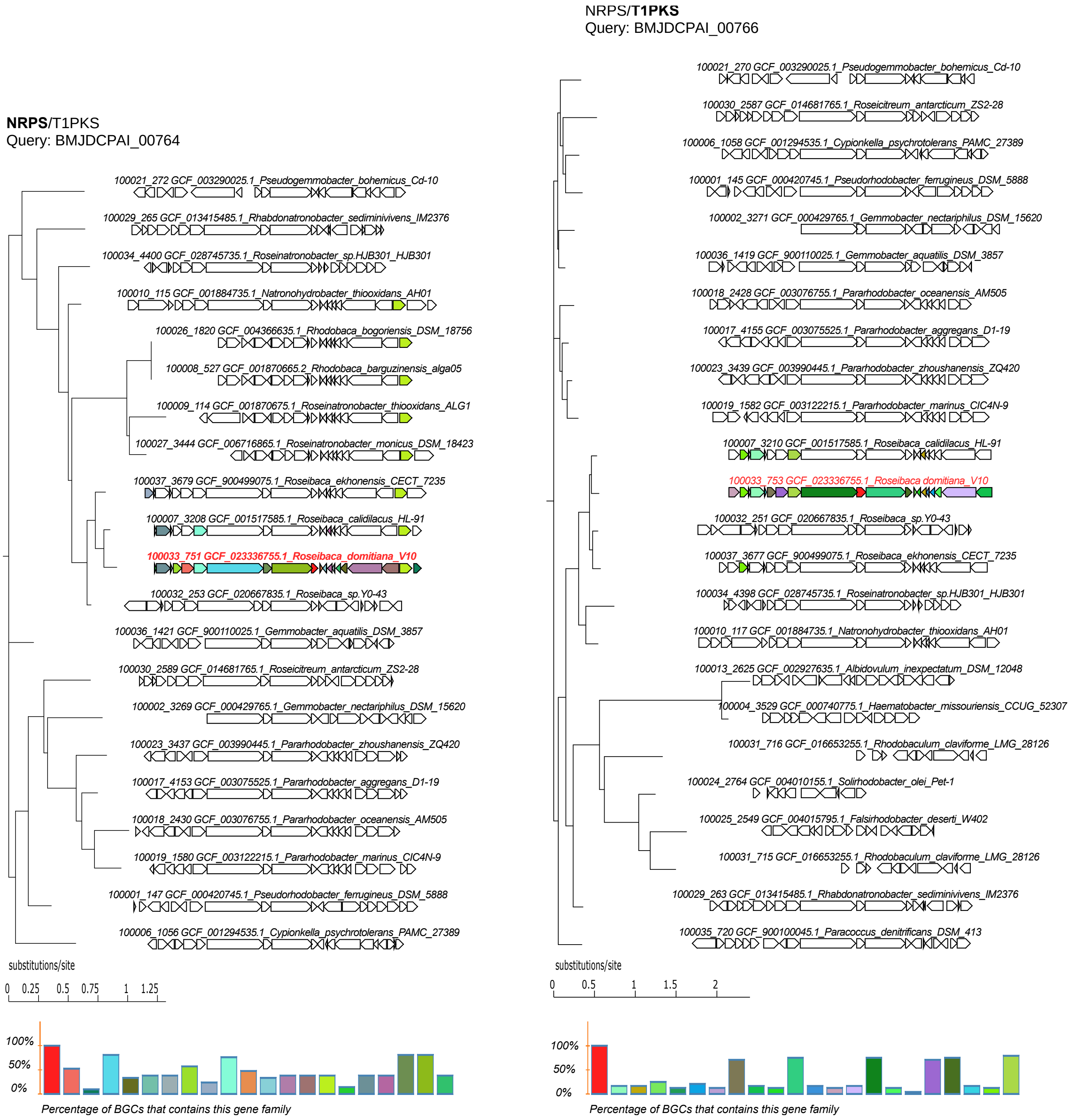

Supplementary Figure S6. Approximate maximum-likelihood reconstructions inferred with CORASON based on NRPS/T1PKS gene cluster of *Roseibaca domitiana* V10^{T}. **(A)** Superfamily tree based on the accessory gene BMJDCPAI_00764 (NRPS) as query. **(B)** Superfamily tree based on the accessory gene BMJDCPAI_00766 (T1PKS) as query. Both phylogenies are showing the copies of similarities of these BGCs in the genome set of species studied. The reduction of intensity of the color or white indicates the low or null homology.

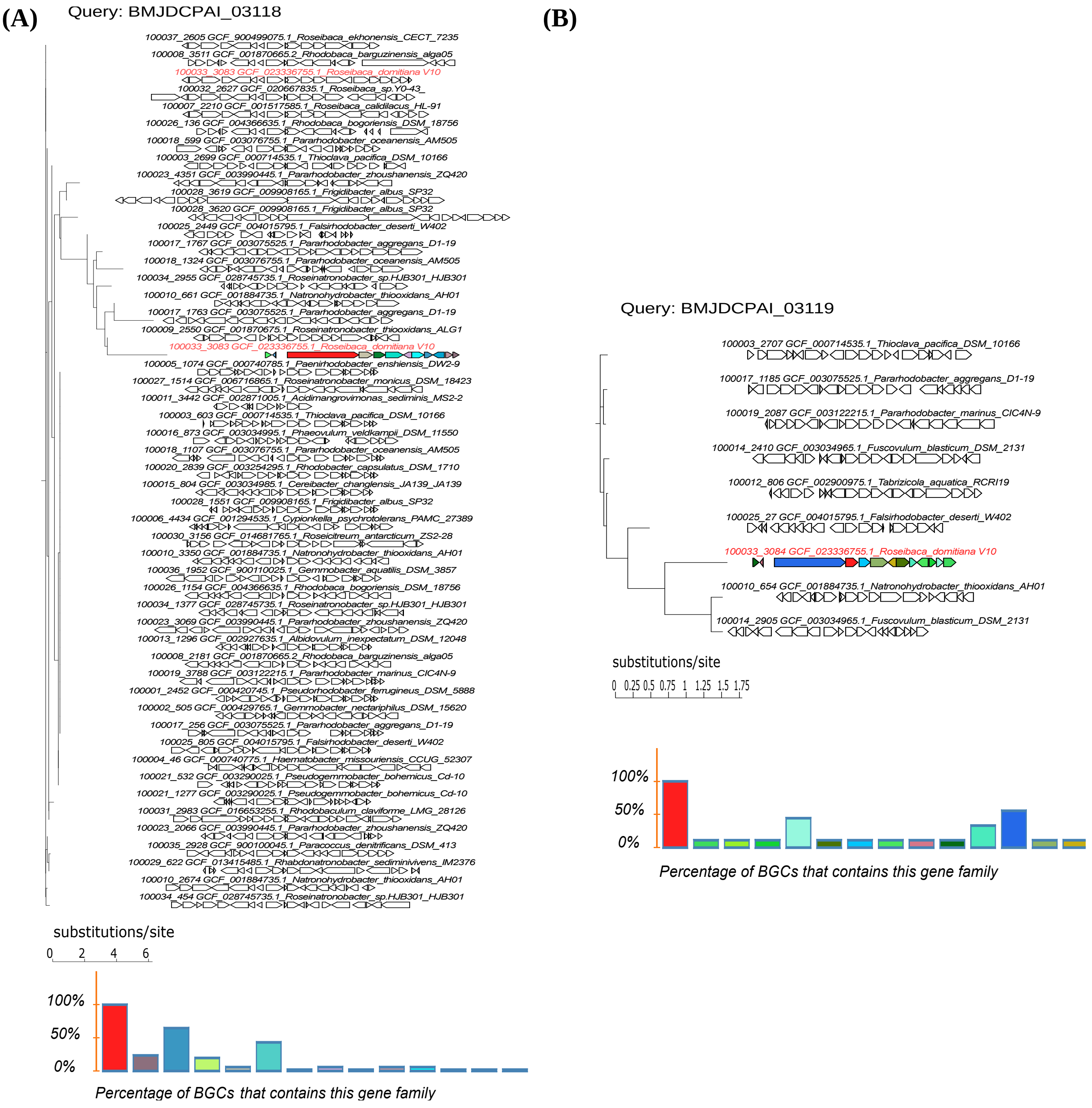

Supplementary Figure S7. Approximate maximum-likelihood reconstructions inferred with CORASON based on T1PKS gene cluster of *Roseibaca domitiana* V10^{T}. **(A)** Superfamily tree based on the accessory gene BMJDCPAI_03118 as query. **(B)** Superfamily tree based on the accessory gene BMJDCPAI_03119 as query. Both phylogenies are showing the copies of similarities of these BGCs in the genome set of species studied. The reduction of intensity of the color or white indicates the low or null homology.

Supplementary Table 1. MIGS of the closely related species to *Roseibaca domitiana* V10^{T}.

Organism	Strain	WGS accession number	Refseq assembly accession number	Genome size (Mb)	Assembly level	GC %	N50	L50	Genes	Protein-coding
Roseibaca domitiana	V10^{T}	JALZWP000000000.1	GCF_023336755.1	3.4	Scaffold	60.5	193,7	6	3,341	3,205
“Roseibaca calidilacus”	HL-91	NZ_FBYC00000000.1	GCF_001517585.1	3.35	Contig	61.8	2,631,035	1	3,285	3,197
Roseibaca ekhonensis	CECT 7235^{T}	NZ_UIHC00000000.1	GCF_900499075.1	3.90	Contig	61.6	73,264	16	3,964	3.888
Roseibaca sp.	Y0-43	JAFMPR000000000.1	GCF_020667835.1	3	Contig	63	299,8	5	3,018	2,945
Roseinatronobacter thiooxidans	ALG1^{T}	NZ_MEHT00000000.1	GCF_001870675.1	3.50	Scaffold	60.3	238,393	6	3,389	3,308
Roseinatronobacter monicus	DSM 18423^{T}	NZ_VFPT00000000.1	GCF_006716865.1	4,73	Contig	59.2	3,772,823	1	4,556	4,457
Roseinatronobacter sp. HJB301	HJB301	JAQZSM000000000.1	GCF_028745735.1	4,8	Scaffold	59.5	149,7	9	4,742	4,559
Rhabdonatronobacter sediminivivens	IM2376^{T}	JACBXS000000000.1	GCF_013415485.1	4.1	Contig	66	90	16	3,720	3,600
Rhodobaca bogoriensis	DSM 18756^{T}	NZ_SORN00000000.1	GCF_004366635.1	3.91	Scaffold	59.0	424,915	3	3,748	3,756
Rhodobaca barguzinensis	alga05^{T}	NZ_CP024899.1	GCF_001870665.2	3.90	Complete	59.0	3,899,419	1	3,707	3,608
“Natronohydrobacter thioxidans”	AH01	MJAP00000000.1	GCF_001884735.1	4.1	Contig	63	310,9	4	3,941	3,840
Roseicitreum antarcticum	ZS2-28^{T}	NZ_CP061498.1	GCF_014681765.1	4.25	Scaffold	63.1	3,537,072	1	3.416	3,250
Pararhodobacter oceanensis	AM505^{T}	NZ_QDKM00000000.1	GCF_003076755.1	4.09	Contig	62.9	547,982	3	3,774	3,664
Pararhodobacter zhoushanensis	ZQ420^{T}	NZ_REEZ00000000.1	GCF_003990445.1	4.84	Scaffold	64.8	388,091	4	4,751	4,658
Pararhodobacter marinus	CIC4N-9^{T}	NZ_QEYD00000000.1	GCF_003122215.1	4.49	Contig	66.7	342,552	6	4,327	4,219
Pararhodobacter aggregans	D1-19^{T}	NZ_QDDR00000000.1	GCF_003075525.1	5.08	Contig	68.2	299,118	6	4,964	4,852
Rhodobaculum claviforme	LMG 28126^{T}	NZ_NHSD00000000.1	GCF_016653255.1	3.49	Scaffold	70.7	14,066	73	3,350	3,208
Alkalilacustris brevis	34079^{T}	NZ_QNVJ00000000.1	GCF_003350345.1	3.48	Contig	65.6	58,978	15	3,259	3,148
Haematobacter missouriensis	CCUG 52307^{T}	NZ_JFGS00000000.1	GCF_000740775.1	3.96	Contig	64.3	106,035	9	3,731	3,612
Rhodobacter capsulatus	DSM 1710^{T}	NZ_QKZO00000000.1	GCF_003254295.1	3.67	Scaffold	66.5	314,919	5	3,547	3,463
Paenirhodobacter enshiensis	DW2-9^{T}	NZ_JFZB00000000.1	GCF_000740785.1	3.44	Contig	66.8	165,802	7	2,856	2,781
Thioclava pacifica	DSM 10166^{T}	NZ_AUND00000000.1	GCF_000714535.1	3.73	Contig	63.9	567,911	3	3,615	3,566
Phaeovulum veldkampii	DSM 11550^{T}	NZ_PZKF00000000.1	GCF_003034995.1	3.26	Contig	65.1	82,916	15	3,273	3,116
Paracoccus denitrificans	DSM 413^{T}	NZ_FNEA00000000.1	GCF_900100045.1	5.19	Scaffold	66.8	162,128	12	5,227	5,097
Frigidibacter albus	SP32^{T}	NZ_JAAAUC000000000.1	GCF_009908165.1	4.19	Contig	67.6	318,621	5	4,013	3,902
Albidovulum inexpectatum	DSM 12048^{T}	NZ_PRDS00000000.1	GCF_002927635.1	3	Contig	64.8	306,579	4	2,966	2,914
Fuscovulum blasticum	DSM 2131^{T}	NZ_PZKE00000000.1	GCF_003034965.1	3.59	Contig	66.5	202,728	7	3,544	3,451
Pseudogemmobacter bohemicus	Cd-10^{T}	NZ_QNHG00000000.1	GCF_003290025.1	5.61	Scaffold	63.2	1,442,964	2	5,416	5,174
Tabrizicola aquatica	RCRI19^{T}	NZ_PJON00000000.1	GCF_002900975.1	3.87	Contig	66.4	692,795	2	3,882	3,791
Cypionkella psychrotolerans	PAMC 27389^{T}	NZ_LGIC00000000.1	GCF_001294535.1	5.08	Contig	60.3	176,187	10	5,084	4,824
Gemmobacter aquatilis	DSM 3857^{T}	NZ_FOCE00000000.1	GCF_900110025.1	3.96	Scaffold	65.1	319,892	4	3,917	3,856
Pseudorhodobacter ferrugineus	DSM 5888^{T}	NZ_ATVN00000000.1	GCF_000420745.1	3.43	Contig	58.5	164,101	8	3,440	3,312
Cereibacter changlensis	JA139^{T}	NZ_PZKG00000000.1	GCF_003034985.1	4.88	Contig	68.0	28,310	49	4,942	4,717
Falsirhodobacter deserti	W402^{T}	NZ_JQHS00000000.1	GCF_004015795.1	2.98	Scaffold	63.4	2,292,967	1	2,980	2,840
Gemmobacter nectariphilus	DSM 15620^{T}	NZ_AUCM00000000.1	GCF_000429765.1	4.52	Scaffold	66.2	317,022	5	4,441	4,309
Acidimangrovimonas sediminis	MS2-2^{T}	NZ_PHSN00000000.1	GCF_002871005.1	5.28	Contig	67.8	240,909	8	4,948	4,831
Solirhodobacter olei	Pet-1^{T}	NZ_RRYH00000000.1	GCF_004010155.1	4.78	Contig	66.8	172,592	9	4,559	4,432

Supplementary Table 2. BGC's count of *Roseibaca domitiana* V10^{T} showing matches with known BGCs present in other organisms.

Region/ Type	From - to	Total length (bp)	Most similar known cluster (Similarity)	Core length Nt	MIBiG Organism hit	Pfam (similarity)
1.1 Terpene	96,093 - 117,127	21,035	Carotenoid (100%)	1035	*Rhodobacter sphaeroides*	PF00494.21 (Squalene/phytoene synthase)
2.1 T3PKS	104,396-145,436	41,041	-	1041	*Streptomyces griseus* subsp. *griseus* NBRC 13350	PF00195.21 (Chalcone and stilbene synthases, N-terminal domain)<br>PF02797.17 (Chalcone and stilbene synthases, C-terminal domain)
2.2 NRPS like, T1PKS	330,790-382,641 (Total hybrid)<br>NRPS<br>350,790-355,316	4527	-	4527	*Streptomyces griseus* subsp. *griseus* NBRC 13350	PF00551.21 (Formyl transferase)<br>PF02911.20 (Formyl transferase, C-terminal domain)<br>PF00501.30 (AMP-binding enzyme)<br>PF00296.22 (Luciferase-like monooxygenase)<br>PF13193.8 (AMP-binding enzymeC-terminal domain)<br>PF00550.27 (Phosphopantetheine attachment site)
	T1PKS<br>356,309-362,641	6333	-	6333	*Monascus aurantiacus*	PF00109.28 (Beta-ketoacyl synthase, N-terminal domain)<br>PF02801.24 (Beta-ketoacyl synthase, C-terminal domain)<br>PF16197.7 (Ketoacyl-synthetase C-terminal extension)<br>PF00698.23 (Acyl transferase domain)<br>PF08659.12 (KR domain)<br>PF14765.8 (Polyketide synthase dehydratase)<br>PF00550.27 (Phosphopantetheine attachment site)<br>PF00975.22 (Thioesterase domain)
3.1 ectoine	48,765-59,151	10,387	Ectoine (80%)	387	*Methylarcula marina*	PF06339.14 (Ectoine synthase)
9.1 RRE-containing	96,516 - 116,791	20,276	-	276	*Paenibacillus dendritiformis* C454	PF05402.14 [Coenzyme PQQ synthesis protein D (PqqD)]
26.1 T1PKS	1 - 26,295	26,295	-	7311	*Solanum lycopersicum*	PF00109.28 (Beta-ketoacyl synthase, N-terminal domain)<br>PF02801.24 (Beta-ketoacyl synthase, C-terminal domain)
